# Organ preservation after neoadjuvant immunotherapy in mismatch repair-deficient colorectal cancer: response assessment, watch-and-wait, and salvage surgery

**DOI:** 10.3389/fonc.2026.1908199

**Published:** 2026-08-25

**Authors:** Nan Yao, Guoshuai Xu, Ning Duan, Fuzhou Han, Wenqiang Li, Jun Qu

**Affiliations:** Department of General Surgery, Aerospace Center Hospital, Beijing, China

**Keywords:** clinical complete response, colorectal cancer, microsatellite instability-high, mismatch repair deficiency, neoadjuvant immunotherapy, organ preservation, rectal cancer, watch-and-wait

## Abstract

Neoadjuvant immune checkpoint blockade has produced high pathological and clinical response rates in mismatch repair-deficient (dMMR) or microsatellite instability-high (MSI-H) colorectal cancer, raising the possibility of response-adapted organ preservation. However, the evidence is anatomically uneven and remains immature for routine surgery avoidance. In dMMR colon cancer, neoadjuvant studies primarily establish drug activity through resection-confirmed pathological and nodal response; reliable criteria for nonoperative assessment have not been validated. In dMMR rectal cancer, clinical complete response (cCR) after PD-1 blockade may support watch-and-wait in highly selected patients because local response can be reassessed using digital rectal examination, endoscopy, pelvic MRI, biopsy when appropriate, systemic imaging, and tumor markers. This narrative review critically appraises the biological rationale, prospective and observational evidence, response-assessment limitations, surgical decision points, surveillance requirements, toxicity, and unresolved controversies surrounding organ preservation after neoadjuvant immunotherapy. We use the term immune-response-adapted surgical deferral to emphasize that surgery remains available for incomplete response, unresolved uncertainty, or regrowth. Current implementation should be confined to experienced multidisciplinary programs with rigorous molecular confirmation, standardized multimodal assessment, reliable follow-up, informed patient participation, and timely salvage capacity. Particular attention is given to immunotherapy-specific uncertainty in defining durable cCR, the limitations of circulating tumor DNA (ctDNA) as a stand-alone local decision tool, hereditary-risk considerations in Lynch syndrome, and the need for location-specific prospective validation.

## Introduction: from surgical radicality to immune-mediated organ preservation

Colorectal cancer remains a major global health burden (1, 2). Curative-intent treatment has traditionally relied on anatomical resection, including oncological colectomy for colon cancer and total mesorectal excision (TME) for rectal cancer (3, 4). Although these operations remain central to cure, their functional consequences create a strong rationale for safe organ preservation, particularly for low rectal tumors. The relevant question is therefore not whether surgery can be avoided at any cost, but whether response-adapted deferral can preserve function without compromising oncological rescue.

Organ preservation was initially developed for selected rectal cancer patients after chemoradiotherapy or total neoadjuvant therapy. The emergence of mismatch repair-deficient (dMMR) or microsatellite instability-high (MSI-H) disease has changed the biological basis of this strategy because immune checkpoint blockade can produce deep regression by restoring pre-existing antitumor immunity. Contemporary rectal cancer guidelines recognize nonoperative management after a carefully established cCR in selected settings, but the evidence supporting immunotherapy-specific watch-and-wait remains substantially less mature than the broader chemoradiotherapy- or TNT-based literature (5–9). Molecular subtype may therefore inform treatment planning, but it does not replace anatomical staging, response verification, or surgical judgment.

Historically, dMMR status was recognized as a molecular feature associated with distinctive clinicopathological characteristics, favorable prognosis in some localized colon cancers, and reduced benefit from fluoropyrimidine-only adjuvant chemotherapy (10, 11). Its therapeutic significance changed substantially after PD-1 blockade demonstrated marked activity in dMMR/MSI-H tumors (12, 13). In metastatic colorectal cancer, pembrolizumab improved progression-free survival compared with chemotherapy in KEYNOTE-177, while nivolumab-based regimens also showed durable activity in this molecular subgroup (14–16). These findings established dMMR/MSI-H status not only as a prognostic marker, but also as a predictive biomarker for immune checkpoint blockade.

The key question has now moved into the curative-intent setting. Short-course checkpoint blockade produces frequent major pathological responses in dMMR colon cancer, whereas prospective rectal cancer cohorts show that selected patients may achieve a cCR and enter nonoperative surveillance (17–20). These observations are clinically compelling but arise from different anatomical sites, endpoints, and evidentiary designs.

**Figure 1:**
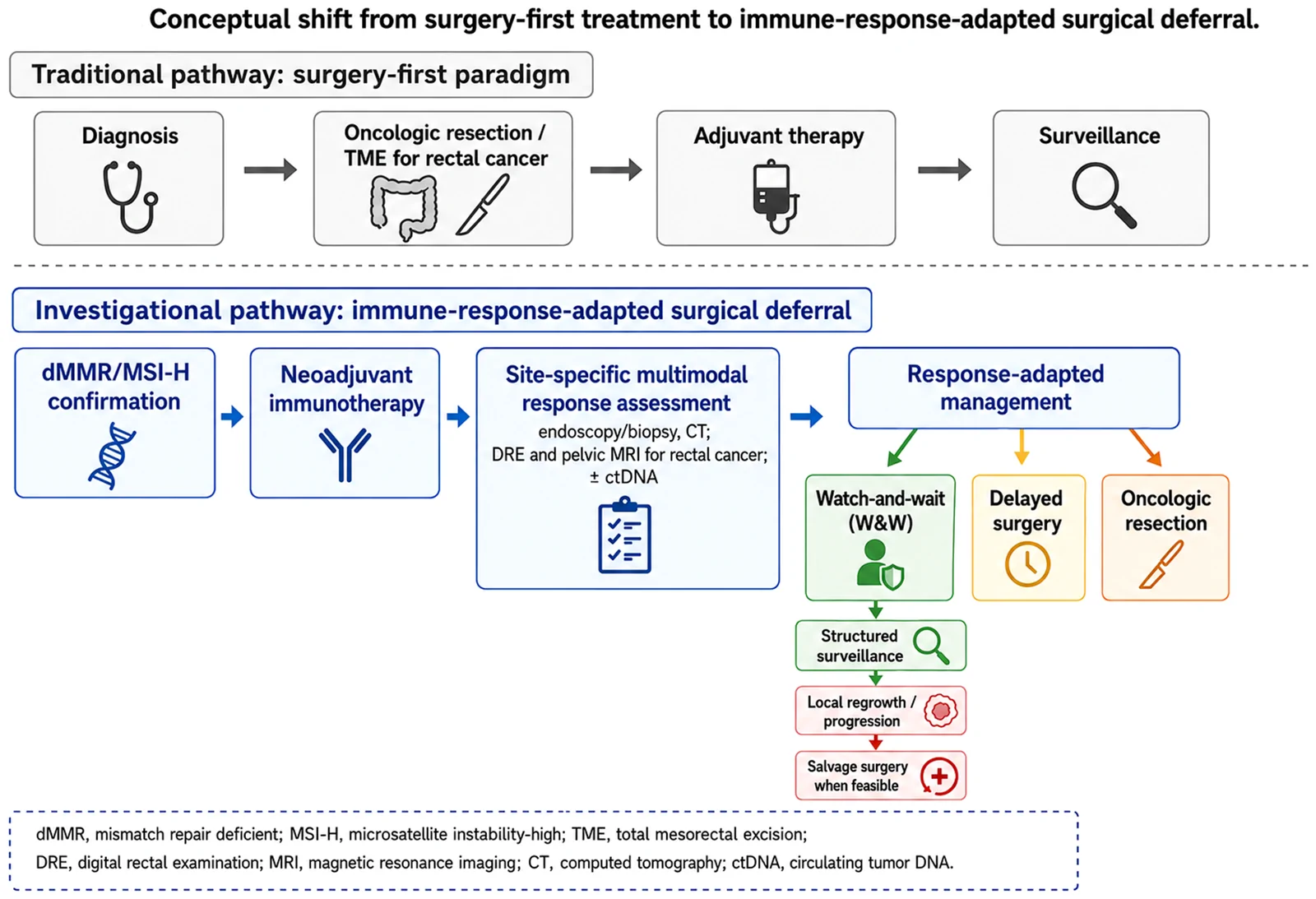
Conceptual shift from surgery-first treatment to immune-response-adapted surgical deferral. The traditional pathway is contrasted with a biomarker-informed investigational framework in which dMMR/MSI-H confirmation leads to neoadjuvant immunotherapy, site-specific multimodal response assessment, and response-adapted management. Management options include protocol-based watch-and-wait with structured surveillance and salvage surgery when feasible if local regrowth or progression occurs, delayed surgery, or oncologic resection. The lower pathway is conceptual and should not be interpreted as an established standard for colon cancer, in which planned resection remains the default outside prospective trials. CT, computed tomography; ctDNA, circulating tumor DNA; dMMR, mismatch repair-deficient; DRE, digital rectal examination; MRI, magnetic resonance imaging; MSI-H, microsatellite instability-high; TME, total mesorectal excision; W&W, watch-and-wait.

These findings create a location-specific surgical dilemma. In dMMR colon cancer, current evidence mainly supports neoadjuvant immunotherapy followed by planned resection, because pathological response and nodal clearance are generally established from the surgical specimen. In dMMR rectal cancer, cCR after PD-1 blockade raises the possibility of deferring TME, but the durability of response, immunotherapy-specific regrowth patterns, and salvage outcomes remain incompletely characterized. Organ preservation should therefore not be interpreted as passive omission of surgery or as a uniform strategy for all dMMR colorectal cancers. In this review, it is framed as immune-response-adapted surgical deferral: an evolving strategy in which surgery is repositioned according to tumor location, response depth, residual uncertainty, surveillance feasibility, patient preference, and salvage capacity. This conceptual framework is illustrated in **Figure 1**.

## Literature search, study selection, and evidence appraisal

This article was designed as a narrative review rather than a systematic review or meta-analysis. PubMed/MEDLINE, Web of Science, Embase, and the Cochrane Library were searched from database inception through July 2026, and ClinicalTrials.gov was examined for relevant ongoing or recently completed studies. Search concepts combined terms for mismatch repair deficiency or microsatellite instability (“dMMR”, “MSI-H”, and “mismatch repair”) with terms for colorectal site and treatment (“colorectal cancer”, “rectal cancer”, “colon cancer”, “neoadjuvant immunotherapy”, “PD-1 blockade”, and “immune checkpoint inhibitor”) and organ-preservation outcomes (“clinical complete response”, “pathological complete response”, “watch-and-wait”, “nonoperative management”, “organ preservation”, “salvage surgery”, “ctDNA”, and “Lynch syndrome”). Reference lists of pivotal trials, guidelines, consensus documents, and major reviews were screened for additional studies.

Eligible evidence addressed nonmetastatic or curative-intent dMMR/MSI-H colorectal cancer, neoadjuvant immune checkpoint blockade, response assessment, watch-and-wait, organ preservation, surveillance, salvage surgery, toxicity, quality of life, or hereditary-risk management. Prospective interventional studies were prioritized over retrospective series and case reports; peer-reviewed full-text publications were prioritized over conference abstracts; and studies reporting long-term oncological, organ-preservation, or salvage outcomes were given greater interpretive weight than studies reporting response rates alone. Conference abstracts were retained only when they provided clinically important information not yet available in full text. Colon and rectal cancer evidence was evaluated separately, and pCR, cCR, organ preservation, and survival were not treated as interchangeable endpoints. No formal risk-of-bias instrument or quantitative certainty grading was applied; accordingly, statements about clinical readiness represent a qualitative narrative appraisal based on study design, sample size, follow-up maturity, endpoint validity, response-assessment feasibility, and availability of salvage data. Preliminary or single-arm findings were interpreted as hypothesis-generating unless supported by convergent prospective evidence.

For the registered-trial landscape, ClinicalTrials.gov was searched through July 21, 2026 using combinations of dMMR/MSI-H, colon or rectal cancer, PD-1/PD-L1 blockade, watch-and-wait, nonoperative management, clinical complete response, treatment duration, and deferred surgery. The trial table includes interventional studies of localized dMMR/MSI-H colorectal cancer that prespecified either an organ-preservation pathway after clinical response or a response-adapted treatment-duration/stopping rule. Mandatory-resection studies without an organ-preservation or response-adapted duration component were excluded from this focused table. Registry status, planned enrollment, regimen, assessment timing, surveillance, and surgery or salvage triggers were extracted from the current record and linked protocol when available; unreported details are identified as not specified rather than inferred. Trial status was rechecked on July 21, 2026.

### Terminology and interpretive scope

For this review, organ preservation is an umbrella objective describing avoidance of radical resection while maintaining curative intent. Watch-and-wait refers specifically to protocol-based active surveillance after a multimodally established cCR; it is not synonymous with absence of treatment or follow-up. Nonoperative management is a broader management category that may include watch-and-wait and other trial-defined strategies that do not involve immediate radical surgery. Local excision is an operative organ-sparing intervention and should not be grouped with watch-and-wait. The phrase surgery omission is used only when describing a study endpoint or a completed treatment course, because at the time of decision-making surgery is more accurately considered deferred and remains available if response is incomplete, assessment is discordant, or regrowth occurs. These definitions are applied separately to rectal and colon cancer because the evidence, functional trade-offs, and ability to assess residual disease differ substantially by anatomical site.

## Biological rationale: why dMMR colorectal cancer is uniquely sensitive to immune checkpoint blockade

Mismatch repair failure involving MLH1, PMS2, MSH2, or MSH6 permits accumulation of insertion-deletion errors and generates the MSI-H phenotype (21, 22). The resulting high mutational and neoantigen burden is commonly accompanied by dense immune infiltration, interferon signaling, cytotoxic T-cell activity, and adaptive expression of inhibitory checkpoints such as PD-1/PD-L1 (23–28). This immunogenic but restrained microenvironment provides the biological basis for checkpoint blockade.

The neoadjuvant setting may enhance this effect because an intact primary tumor and draining lymphatic network provide abundant antigen during treatment. Preclinical and translational data suggest that checkpoint blockade before resection can expand tumor-reactive T-cell clones and generate systemic immune memory (29). This mechanism is consistent with the rapid pathological regression observed in colon cancer and the deep clinical regression reported in rectal cancer, but response is not uniform across patients or compartments.

dMMR/MSI-H status strongly enriches for benefit but does not guarantee eradication. Primary or acquired resistance may involve impaired antigen presentation, HLA or beta-2-microglobulin alterations, JAK-STAT pathway disruption, immune exclusion, or intratumoral heterogeneity. Moreover, regression of the mucosal primary does not prove clearance of deeper wall or nodal disease. Biology therefore supports neoadjuvant immunotherapy, whereas the safety of organ preservation must be established through location-specific response assessment and long-term clinical outcomes.

## Clinical evidence for neoadjuvant immunotherapy in dMMR colorectal cancer

### Colon cancer: compelling surgery-confirmed activity, unproven nonoperative safety

The strongest surgery-confirmed evidence comes from localized dMMR colon cancer. NICHE and NICHE-2 demonstrated rapid major pathological responses, including frequent pCR, after short-course nivolumab plus ipilimumab before planned colectomy (17, 18). These studies establish biological activity and perioperative feasibility; they do not validate colectomy omission because residual mural and nodal disease was determined from the surgical specimen.

Evidence outside selected prospective cohorts is more heterogeneous. A multicenter real-world study reported lower pathological response and greater serious toxicity than landmark trials, while a systematic review identified substantial variation in regimens, response definitions, and follow-up (30, 31). The colon-inclusive NEOCAP phase II study provides direct but still preliminary nonoperative evidence: among 52 evaluable patients treated with camrelizumab plus apatinib, 28 (54%) achieved cCR and 24 entered W&W, including 20 patients with colon or multiple colorectal primaries; median follow-up was 16.4 months and no regrowth was reported during that short interval (32). These findings establish feasibility in selected patients, not the diagnostic accuracy or long-term oncological safety required for routine colectomy omission.

### Rectal cancer: emerging organ-preservation evidence with limited long-term validation

Rectal cancer presents a different clinical opportunity because the primary site is repeatedly accessible and avoidance of TME may yield substantial functional benefit. In the dostarlimab program, selected patients with dMMR locally advanced rectal cancer achieved cCR after PD-1 blockade and entered nonoperative surveillance; subsequent prospective reporting broadened this experience (19, 20). The cohorts remain nonrandomized, concentrated in expert programs, and limited in size and follow-up relative to the time required to establish durable organ preservation.

Other prospective studies, including PICC and NEOPRISM-CRC, confirm sensitivity across checkpoint inhibitors and treatment schedules, but most used resection-confirmed pathological endpoints rather than durable nonoperative control (33, 34). Comparisons of response percentages are therefore confounded by site, stage, regimen, treatment duration, mandatory surgery, and endpoint definition. The evidence supports high neoadjuvant activity in colon cancer and emerging protocol-based W&W in selected rectal cancer—not a uniform nonoperative strategy across colorectal sites.

The evidence base for routine organ preservation remains immature: most studies are phase II, single-arm, and selected; immunotherapy-specific cCR definitions vary; randomized comparisons are lacking; and long-term regrowth, distant relapse, and salvage outcomes are incompletely characterized. Clinical readiness should therefore be judged by durable cCR, organ-preservation survival, salvageability, functional outcomes, and late toxicity rather than initial response alone. **Tables 1**, **2** summarize this location- and endpoint-specific interpretation, while the principal clinical studies of neoadjuvant immunotherapy are summarized in **Supplementary Table 1**.

**Table 1:** Differences between rectal and colon cancer in organ preservation after immunotherapy.

Dimension	Rectal cancer	Colon cancer	Clinical implication
Organ preservation value	High functional value, especially for low rectal tumors, because avoidance of TME may prevent permanent stoma, low anterior resection syndrome, and urinary or sexual dysfunction.	Usually lower functional gain than rectal preservation, because colectomy is often better tolerated and does not usually cause the same pelvic functional burden.	The clinical rationale for organ preservation is generally stronger in rectal cancer, but implementation still requires selected patients, expert assessment, and prospective validation.
Assessment method	Multimodal local assessment is feasible using digital rectal examination, endoscopy, biopsy when appropriate, pelvic MRI, diffusion-weighted imaging, and systemic restaging.	Local assessment is less mature. Colonoscopy can assess mucosal healing, but it cannot reliably exclude transmural or nodal residual disease; CT/MRI are less validated for cCR determination.	cCR-based decision-making is more clinically actionable in rectal cancer; colon cancer generally still requires resection to confirm pathological response.
W&W experience	Extensive experience exists after chemoradiotherapy/TNT; early dMMR immunotherapy studies provide emerging, not definitive, support for selected patients with cCR.	Very limited experience. Most neoadjuvant immunotherapy studies in dMMR colon cancer still use surgery to confirm pCR or major pathological response.	Immunotherapy-based W&W may be considered selectively for rectal cancer; colon cancer W&W remains investigational.
Local regrowth detection	Early local regrowth can often be detected by symptoms, DRE, endoscopy, and pelvic MRI during intensive surveillance.	Detection is more difficult because subtle mural or nodal residual disease is harder to monitor repeatedly and reliably outside surgical pathology.	Surveillance infrastructure is a key reason why organ preservation is more mature in rectal cancer.
Salvage feasibility	Salvage TME or local surgery may preserve curative intent when regrowth is detected early, although immunotherapy-specific salvage outcomes require longer follow-up.	Salvage colectomy may be feasible if localized disease is detected, but delayed recognition of residual or nodal disease could compromise oncologic safety.	Any organ-preservation program requires realistic access to timely salvage, although immunotherapy-specific salvage outcomes remain uncertain.
Current evidence maturity	Emerging and clinically important, but based mainly on selected single-arm cohorts with limited long-term follow-up.	Biologically promising but clinically immature; high pCR rates after neoadjuvant immunotherapy do not yet validate routine omission of colectomy.	Future trials should evaluate rectal and colon cancer separately, with different endpoints and surveillance rules.

cCR, clinical complete response; DRE, digital rectal examination; dMMR, mismatch repair-deficient; MRI, magnetic resonance imaging; pCR, pathological complete response; TME, total mesorectal excision; TNT, total neoadjuvant therapy; W&W, watch-and-wait.

The table is intended to support conceptual comparison in a narrative review rather than to define formal treatment guidelines.

**Table 2:** Evidence strength and clinical readiness of organ preservation after neoadjuvant immunotherapy.

Clinical setting	Evidence maturity	Current implication	Key uncertainty	Clinical readiness
dMMR colon cancer after neoadjuvant immunotherapy	High surgery-confirmed pathological response evidence; limited nonoperative evidence	Supports neoadjuvant immunotherapy before planned colectomy in selected settings	Whether colectomy can be safely omitted and how nodal clearance can be verified without resection	Low for routine W&W; investigational outside trials
dMMR rectal cancer with robust cCR	Emerging prospective organ-preservation evidence; selected cohorts, nonrandomized designs, and limited long-term follow-up	May support protocol-based W&W in selected patients within expert MDT programs	Long-term durability, local regrowth biology, and immunotherapy-specific salvage outcomes	Conditional and limited; specialist programs or prospective protocols
dMMR rectal cancer with near-cCR	Indirect and evolving evidence; usually extrapolated from rectal W&W and early immunotherapy cohorts	Supports time-limited reassessment or protocol-defined management rather than open-ended observation	Optimal reassessment interval, number of cycles, and role of local excision or additional immunotherapy	Limited; requires predefined MDT protocol
Residual or progressive disease after immunotherapy	Conventional oncologic evidence remains strongest for resection when response is incomplete	Radical surgery generally remains the reference oncologic pathway	Whether selected patients benefit from additional immunotherapy before surgery	Established role for surgery when residual disease is convincing
Colon cancer watch-and-wait after apparent complete response	Very limited evidence; validated local cCR criteria are lacking	Not supported as routine care	Inability to reliably exclude transmural or nodal residual disease without colectomy	Investigational; prospective trials or exceptional individualized circumstances
Lynch syndrome-associated dMMR colorectal cancer	Established hereditary-risk evidence; very limited organ-preservation-specific evidence	Requires integration of tumor response with hereditary-risk counseling and long-term surveillance planning	Optimal balance between segmental preservation, metachronous cancer risk, function, and patient preference	Individualized; requires hereditary-risk planning

cCR, clinical complete response; dMMR, mismatch repair-deficient; MDT, multidisciplinary team; near-cCR, near-complete response; W&W, watch-and-wait.

This table provides a narrative synthesis of current evidence strength and clinical readiness across different organ-preservation scenarios after neoadjuvant immunotherapy. The categories reflect the authors’ interpretation based on available clinical evidence, response-assessment feasibility, surveillance reliability, and salvage-surgery considerations, and should not be interpreted as formal treatment guidelines.

## Response assessment after neoadjuvant immunotherapy: cCR, pCR, and the problem of discordance

Response assessment is the central bottleneck between neoadjuvant immunotherapy and organ preservation. pCR is established after resection by demonstrating no viable tumor in the treated primary site or sampled regional nodes. cCR is an inferential, time-dependent clinical state based on the absence of detectable disease across examination, endoscopy, imaging, and other restaging tests. It is therefore a management threshold rather than a pathological diagnosis, and its false-negative consequences are greatest when surgery is deferred.

### Rectal cancer: multimodal cCR assessment and immunotherapy-specific uncertainty

Rectal cCR criteria were developed mainly after chemoradiotherapy or total neoadjuvant therapy. They combine no palpable tumor on digital rectal examination, a flat white scar or mucosal normalization without ulceration or nodularity on endoscopy, and fibrotic low-signal change without suspicious intermediate T2 signal, restricted diffusion, or residual nodes on pelvic MRI (5–8, 35–38). Concordance across modalities is more informative than any single test, but these criteria have not been fully validated after immunotherapy.

Checkpoint blockade may produce immune infiltration, edema, ulcer healing, fibrosis, mucinous change, delayed regression, and heterogeneous clearance that complicate interpretation. Endoscopy and superficial biopsy cannot exclude deeper mural or nodal disease; MRI may be confounded by treatment effect and reader variability; CT is primarily a systemic restaging tool; and CEA lacks sufficient sensitivity or specificity for local decisions (35–38). A negative biopsy should not overrule suspicious examination, endoscopic, or MRI findings.

Timing and discordance require prespecified management. When the patient is stable, short-interval reassessment by the same expert teams may distinguish delayed regression from persistent disease; progressive or unresolved discordance should lower the threshold for tissue clarification or surgery. Durable cCR should denote serially concordant absence of detectable disease, with separate reporting of treatment completion, first cCR, confirmation, and sustained response. No immunotherapy-validated interval or minimum duration currently defines durability.

### Colon cancer: tentative investigational response frameworks

No validated colon-specific cCR framework exists. Published and registered studies use heterogeneous investigator-derived constructs. NEOCAP assessed response after four and eight cycles using site-adapted radiology and endoscopy and offered W&W after cCR; NAIO defines major clinical response after 18 weeks by an asymptomatic primary tumor, no metastatic disease, and stable or declining ctDNA; and PREMICES requires two successive response assessments and uses scheduled CT plus colonoscopy with biopsy in its nonoperative arm (32, 39–42). WINDOW uses at least four cycles of tislelizumab and requires two consecutive tumor-informed MRD-negative samples before W&W, whereas its linked protocol also incorporates RECIST imaging, colonoscopy with biopsy, tumor markers, and site-specific examination (43). In a 2025 conference abstract, 24 patients were reported, 20 entered W&W, and no recurrence was observed at a median follow-up of 20.3 months; these preliminary, non-peer-reviewed abstract data do not establish long-term safety or validate ctDNA as a stand-alone decision rule (44). These are protocol eligibility or management rules—not validated diagnostic criteria—and they differ in whether local mucosal, transmural, nodal, and molecular clearance must be demonstrated.

Across these investigational frameworks, recurring domains include resolution of obstruction, bleeding, or other tumor-related symptoms; no radiological progression or distant disease; marked endoscopic regression or disappearance of visible tumor; biopsy without viable tumor when required; stable or declining CEA; and ctDNA/MRD decline or clearance. However, the evidence supporting each domain is uneven. Superficial biopsy cannot exclude transmural or nodal tumor, CT has not been validated to exclude microscopic bowel-wall disease, radiological CR under RECIST is not synonymous with sterilization of the primary and regional nodes, and ctDNA negativity may reflect low shedding. Serial confirmation and multidisciplinary review reduce but do not eliminate this uncertainty; no current combination has a validated negative predictive value sufficient to replace colectomy outside a prospective trial.

Accordingly, response assessment must be location-specific. A concordant rectal cCR may serve as an entry criterion for protocol-based W&W, whereas an apparent colon response remains investigational and should not be assumed to represent pCR. Prospective studies should correlate each proposed criterion with resection pathology or sufficiently long nonoperative outcomes and should report false-negative local and nodal disease explicitly.

## Watch-and-wait after immunotherapy: who may be suitable?

Immunotherapy-based W&W is an evolving rectal cancer strategy rather than an established colorectal standard. It may be considered for selected patients with molecularly confirmed dMMR/MSI-H rectal cancer, no distant disease, concordant and confirmed multimodal cCR, informed acceptance of residual uncertainty, reliable adherence, and immediate access to expert surveillance and salvage surgery (9, 19, 20, 35–38, 45–48). When these conditions are not met, surgery may provide the safer curative pathway.

Conventional W&W cohorts show that many rectal regrowths occur early and can often be salvaged when detected promptly (6, 45–48). These data inform surveillance design but are not direct proof for immunotherapy-treated disease, in which the timing of regrowth and outcomes of salvage remain less mature.

W&W is inappropriate for progressive or convincing residual disease, uncontrolled symptoms, threatened obstruction or perforation, unreliable follow-up, or unavailable salvage. Near-cCR requires a predefined reassessment window rather than open-ended observation. For colon cancer, nonoperative management should remain confined to prospective trials or exceptional circumstances in which standard surgery is infeasible and uncertainty is explicitly documented.

## Surgical decision-making after response: immediate surgery, delayed surgery, local excision, or nonoperative management?

After immunotherapy, the choice among planned radical surgery, time-limited reassessment, selected local excision, and W&W should integrate anatomical site, baseline nodal and margin risk, response category, concordance, patient priorities, and salvageability. These pathways differ in both purpose and evidentiary support.

Radical surgery remains appropriate for residual or progressive disease, high-risk anatomy, persistent symptoms, or unresolved assessment uncertainty. It also remains the default after neoadjuvant immunotherapy for operable colon cancer outside a trial. Deferral should not allow obstruction, perforation, systemic progression, or loss of a technically curative window.

### Near-complete response: the key grey zone between organ preservation and surgery

Near-cCR is a time-limited state rather than treatment success or failure. Selected stable rectal cancer patients may undergo short-interval reassessment or protocol-defined additional therapy, with the interval and surgical triggers documented in advance. Persistent or progressive abnormality should prompt resection rather than indefinite extension.

Local excision has a narrower investigational role for small superficial rectal abnormalities when nodal and mesorectal assessment is otherwise reassuring. It may clarify pathology while preserving the rectum, but cannot reliably address occult nodal disease. A confirmed cCR may instead lead to W&W, with prompt restaging and consideration of salvage resection if regrowth develops. Immunotherapy-specific salvage resectability, morbidity, and long-term control require prospective reporting.

## Practical MDT workflow for implementation

**Figure 2:**
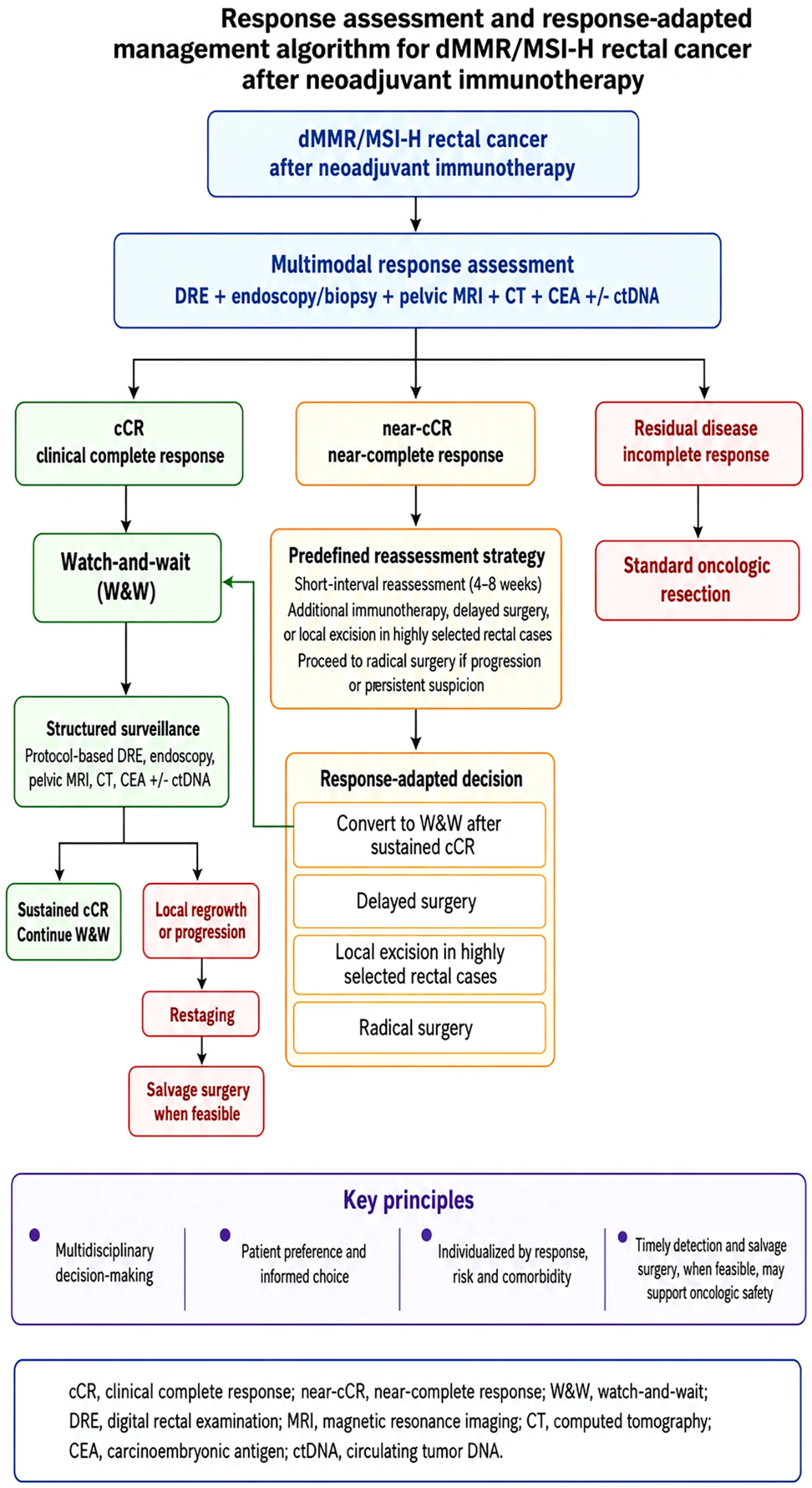
Response assessment and response-adapted management algorithm for dMMR/MSI-H rectal cancer after neoadjuvant immunotherapy. After multimodal assessment in a specialist multidisciplinary program, patients are categorized as having cCR, near-cCR, or residual/incomplete response. A concordant and sustained cCR may support protocol-based watch-and-wait. Near-cCR should prompt a predefined short-interval reassessment strategy, after which selected patients may enter watch-and-wait following sustained cCR, undergo delayed surgery or highly selected local excision, or proceed to radical surgery. Residual/incomplete response generally warrants standard oncologic resection. Only local regrowth or progression during watch-and-wait proceeds to restaging and consideration of salvage surgery when feasible. This algorithm has not been validated for colon cancer, for which planned colectomy remains standard outside a clinical trial. cCR, clinical complete response; CEA, carcinoembryonic antigen; CT, computed tomography; ctDNA, circulating tumor DNA; dMMR, mismatch repair-deficient; DRE, digital rectal examination; MRI, magnetic resonance imaging; MSI-H, microsatellite instability-high; W&W, watch-and-wait.

A response-adapted pathway begins with validated dMMR/MSI-H confirmation, appropriate germline evaluation, high-quality baseline anatomical and systemic staging, and documentation of symptoms and surgical options. Before treatment, the MDT should define site-specific restaging, response categories, reassessment intervals, and triggers for surgery or salvage. **Figure 2** summarizes the proposed rectal pathway; its cCR branches should not be extrapolated to colon cancer, for which planned colectomy remains the default outside trials.

## Surveillance strategy during organ preservation

Surveillance is the active treatment component of organ preservation. During the first two years after rectal cCR, frequent digital rectal examination, endoscopy, and pelvic MRI should evaluate local regrowth, with periodic CT of the chest, abdomen, and pelvis and longitudinal CEA for systemic relapse. Exact intervals vary among programs and should be protocolized; the key requirements are consistent modalities, experienced interpretation, rapid review of discordant findings, and timely salvage access (6, 7, 35–38, 45–48). An illustrative surveillance framework is provided in **Supplementary Table 2**.

Intervals may lengthen after the highest-risk early period, but structured follow-up should continue for at least five years and be individualized by baseline stage, initial nodal risk, treatment duration, response kinetics, hereditary risk, and adherence. New bleeding, pain, altered bowel habit, weight loss, or obstructive symptoms require assessment outside the routine schedule.

ctDNA is an investigational adjunct whose sensitivity varies with burden, site, biological shedding, assay design, and sampling time (49–51). A negative result cannot exclude small-volume disease confined to the rectal wall or mesorectal nodes; a positive result does not localize disease. No threshold or ctDNA-guided rule is validated for omitting rectal or colon surgery after immunotherapy. It should therefore complement, not replace, anatomical and clinical assessment, with prospective trials reporting assay, time points, false-negative local failures, and management consequences.

## Toxicity, quality of life, and patient-centered outcomes

The aim of organ preservation is improved patient-centered outcome without loss of oncological safety. Checkpoint blockade can cause acute or delayed immune-related toxicity, including colitis, hepatitis, pneumonitis, endocrinopathies, dermatological events, arthritis, and rare neurological or cardiac complications (52, 53). Trials should report cumulative exposure, treatment discontinuation, hospitalization, long-term hormone replacement, and late toxicity rather than assuming immunotherapy is functionally neutral.

The functional argument is strongest for rectal cancer. TME, especially for low tumors, may result in permanent or temporary stoma, low anterior resection syndrome with urgency, clustering, fragmentation or incontinence, and urinary or sexual dysfunction (54, 55). Avoiding TME may improve bowel function and quality of life in selected patients, although W&W itself can generate anxiety and repeated-procedure burden; prospective cohorts should measure both benefits and harms (56).

Shared decision-making should elicit how each patient values functional preservation, definitive pathology, recurrence uncertainty, immune toxicity, surveillance burden, and possible salvage. Success should be evaluated with disease-free and overall survival, local regrowth-free and organ-preservation survival, stoma-free survival, validated bowel/urinary/sexual function, quality of life, treatment burden, and patient-reported outcomes—not cCR or surgery avoidance alone (57, 58).

## Discussion: current controversies and unanswered questions

The central uncertainty is whether clinically measured regression can support durable organ preservation without compromising cure. Colon studies largely validate drug activity through resection pathology, whereas rectal nonoperative studies depend on serial cCR and salvageable follow-up (17–20, 30, 31, 33–38, 59). These endpoints are not interchangeable. High colon pCR rates should motivate validation of response prediction, not immediate adoption of W&W.

Three evidence gaps are particularly consequential: no validated colon cCR framework; no immunotherapy-specific definition or confirmation interval for durable rectal cCR; and limited long-term data on regrowth, distant relapse, and salvage. Optimal checkpoint inhibitor, combination, duration, and stopping rule also remain undefined. Trial protocols now range from brief preoperative regimens to six months or longer of therapy, underscoring that schedule choice is still an empirical research question rather than a clinical standard.

Lynch syndrome adds a lifelong hereditary-risk horizon. Response of the index tumor does not remove gene-dependent risks of metachronous colorectal or extracolonic cancer. When indicated, germline clarification, genetic counseling, cascade testing, and gene-, age-, sex-, and family-history-informed surveillance should therefore precede or accompany a definitive organ-preservation plan (60–63).

The trade-off is site-specific. In Lynch-associated colon cancer, preserving the index segment must be weighed against metachronous risk and the conventional choice between segmental and extended colectomy. In rectal cancer, avoiding TME may offer greater functional benefit, but surveillance of the remaining colon and extracolonic organs remains necessary. Current cohorts cannot establish whether cCR durability differs between germline and sporadic dMMR disease; decisions should therefore integrate hereditary risk rather than generalize from tumor response alone.

## Future directions: toward risk-adapted organ preservation trials

Future trials should be location-specific and pathway-focused. Rectal studies need prespecified cCR and near-cCR definitions, confirmation time points, surveillance schedules, and salvage triggers. Colon studies must validate whether any multimodal construct can exclude residual mural and nodal disease with sufficient accuracy for nonoperative management. Comparative cohorts, independent response review, and long follow-up are essential.

Endpoints should include local regrowth, distant relapse, organ-preservation survival, salvage R0 resection, postoperative morbidity, stoma-free survival, immune toxicity, function, quality of life, and patient-reported burden. Biomarker work should prespecify assays and sampling times and should evaluate incremental value beyond anatomy rather than substituting molecular results for local assessment. Any multivariable response model should be transparently developed, validated, and reported (64).

### Registered trials addressing treatment duration and W&W protocols

The predefined registry search identified eight organ-preservation or response-adapted duration studies (**Table 3**), including the multicenter AZUR-1 registrational phase II study of dostarlimab in untreated dMMR/MSI-H locally advanced rectal cancer (65). In July 2026, an official sponsor announcement reported that AZUR-1 had met its primary objective of sustained cCR at 12 months; 154 participants received treatment. However, numerical response data and full peer-reviewed results were not yet publicly available, and the finding should therefore be interpreted cautiously (66). Treatment exposure ranges from four 3-weekly sintilimab cycles in a completed rectal cohort to at least four and up to eight response-guided tislelizumab cycles in WINDOW, six dostarlimab doses over 18 weeks in NAIO, eight pembrolizumab cycles with possible extension to 17 in PREMICES, and nine dostarlimab doses over six months in the rectal dostarlimab programs (39–43, 65, 67, 68). NEOCAP assessed camrelizumab plus apatinib after four and eight cycles. This heterogeneity precludes cross-trial inference that any cycle number is optimal.

**Table 3:** Registered interventional trials meeting predefined criteria for treatment-duration or organ-preservation pathways after neoadjuvant PD-1 blockade.

Trial/site; status*	Design/sample size	Regimen/duration	Response assessment/timing	W&W eligibility	Surgery/salvage trigger	Surveillance information
NCT04165772/rectum; active, published cohort	Phase II, single arm/expansion ongoing	Dostarlimab 500 mg q3w ×9/6 months	DRE, endoscopy/biopsy, pelvic MRI and CT/PET at protocol time points; cCR assessed during and after therapy	Concordant cCR after PD-1 blockade	Incomplete response or later regrowth: protocol-directed additional treatment and/or surgery	Intensive serial endoscopy, MRI, examination and systemic imaging; exact schedule per protocol
NCT05723562 AZUR-1/rectum; active, not recruiting	Global multicenter phase II, single arm/154 enrolled	Dostarlimab 500 mg q3w ×9/6 months	DRE, endoscopy, pelvic MRI and systemic imaging; cCR assessed after therapy, with 12-month cCR by independent central review as a primary endpoint	cCR after dostarlimab permits nonoperative management	Incomplete response or recurrence: protocol-directed oncologic treatment, including surgery when indicated	Close nonoperative surveillance for recurrence; complete public schedule not specified
NCT04304209/rectum; closed, published	Phase II dMMR cohort/17 treated	Sintilimab q3w ×4/12 weeks	DRE, endoscopy, rectal MRI, CT and biopsy; reassessment after four cycles	cCR patients could choose W&W	Non-cCR: surgery; suspected regrowth: restaging and salvage	Prospective W&W follow-up reported; long-term immunotherapy-specific salvage data limited
NCT04715633 NEOCAP/colon and rectum; completed, published	Phase II, single arm/53	Camrelizumab plus apatinib; response checks after cycles 4 and 8	CT for colon, pelvic MRI for rectum, endoscopy and biopsy; site-adapted cCR	cCR after treatment; patient choice	Non-cCR/inadequate response: site-specific therapy or surgery	Protocol follow-up; published median follow-up 16.4 months, insufficient for durable safety
NCT05239546 NAIO/colon; recruiting	Phase II, single arm/registry-planned enrollment	Dostarlimab q3w ×6/18 weeks; continued treatment in eligible responders	Symptoms, metastatic staging and ctDNA after induction; colonoscopy/biopsy and imaging per protocol	Major clinical response: asymptomatic primary, no metastases, stable/declining ctDNA	Nonresponse, progression or failure of protocol criteria: colectomy	Registry specifies structured nonoperative follow-up; detailed universal cCR scar criteria not established
NCT06262581/colon and rectum; unknown status (last verified February 2024)	Phase II, single arm/40	Tislelizumab q3w; protocol-defined neoadjuvant course	Imaging and endoscopic/clinical assessment; detailed confirmation interval incompletely specified publicly	Patients qualified as cCR may enter W&W	Non-cCR or progression: planned surgery; recurrence: MDT-directed treatment	Public record does not fully specify all W&W intervals
NCT06477991 WINDOW/colon and rectum; recruiting; preliminary conference report	Single-center prospective phase II/registry-planned 22; abstract-reported 24	Tislelizumab 200 mg q3w, ≥4 cycles; response-guided to 8	Tumor-informed MRD after cycle 4 and sequentially; RECIST imaging, colonoscopy/biopsy, markers and site-specific examination	Two consecutive MRD-negative results	MRD still positive after cycle 8: surgery; conversion to positive or clinical recurrence: reassessment	Abstract: 20 entered W&W, with no recurrence at median 20.3 months; preliminary conference evidence. Registry: MRD at months 1, 4, 7, 13, 19, 25 and 37, with protocolized clinical and imaging follow-up
NCT06646445 PREMICES/colon; not yet recruiting	Multicenter randomized phase II/84	Pembrolizumab 200 mg q3w ×8; responders may extend to 17 cycles	CT and two successive colonoscopies with biopsy; strategy success assessed at 6 months	Complete response without tumor on serial assessment/biopsy in experimental strategy	Progression, persistent tumor on biopsy or decision for primary-tumor surgery constitutes strategy failure	Scheduled CT and colonoscopy/biopsy; 24-month strategy success and salvage-related outcomes planned

cCR, clinical complete response; CT, computed tomography; ctDNA, circulating tumor DNA; DRE, digital rectal examination; MDT, multidisciplinary team; MRD, molecular residual disease; MRI, magnetic resonance imaging; PD-1, programmed cell death protein 1; PET, positron emission tomography; q3w, every 3 weeks; RECIST, Response Evaluation Criteria in Solid Tumors; W&W, watch-and-wait.

*Trial status reflects the ClinicalTrials.gov records accessed on July 21, 2026. NCT06262581 was listed as having an unknown status because its record had not been verified within the preceding two years. “Not specified” or equivalent wording indicates that sufficient information was not available in the public registry record or linked protocol. Sample sizes represent planned enrollment, actual enrollment, or abstract-reported enrollment, as explicitly indicated. Registry protocols and preliminary conference abstracts do not constitute evidence of established efficacy, and the eligibility and assessment criteria summarized here should not be interpreted as validated cCR standards or formal clinical recommendations.

Response and rescue rules are similarly inconsistent. Rectal studies generally combine digital examination, endoscopy, MRI, and systemic imaging, but confirmation intervals and durable-cCR definitions differ or remain incompletely reported. Colon-inclusive protocols variously rely on symptoms, CT, colonoscopy/biopsy, RECIST response, or serial ctDNA/MRD; WINDOW specifies surgery when MRD remains positive after eight cycles, while PREMICES defines failure through progression, persistent tumor on biopsy, or primary-tumor surgery (39–43, 67, 68). Protocols provide much less mature evidence on regrowth localization, salvage R0 rates, and morbidity than on initial response. The absence of a common treatment duration, cCR definition, surveillance schedule, and salvage trigger is therefore a principal finding rather than a basis for choosing among regimens.

## Conclusions

Neoadjuvant immunotherapy has demonstrated marked activity in dMMR/MSI-H colorectal cancer, but organ-preservation evidence is anatomically uneven. Colon cancer data primarily support surgery-confirmed pathological efficacy; proposed nonoperative response criteria remain investigational. In selected rectal cancer patients, a confirmed cCR may support W&W within expert multidisciplinary programs, provided that uncertainty, surveillance, and timely salvage are integral to the plan. The immediate research priority is not broader surgery omission, but prospective validation of site-specific response criteria, treatment duration, durable control, salvage outcomes, ctDNA utility, and hereditary-risk-informed care.

## References

[1] Sung H Ferlay J Siegel RL Laversanne M Soerjomataram I Jemal A . Global cancer statistics 2020: GLOBOCAN estimates of incidence and mortality worldwide for 36 cancers in 185 countries. CA Cancer J Clin. (2021) 71:209–49. doi: 10.3322/caac.21660 33538338

[2] Arnold M Abnet CC Neale RE Vignat J Giovannucci EL McGlynn KA . Global burden of 5 major types of gastrointestinal cancer. Gastroenterology. (2020) 159:335–349.e15. doi: 10.1053/j.gastro.2020.02.068 32247694 PMC8630546

[3] Heald RJ Husband EM Ryall RDH . The mesorectum in rectal cancer surgery-the clue to pelvic recurrence? Br J Surg. (1982) 69:613–6. doi: 10.1002/bjs.1800691019 6751457

[4] Sauer R Becker H Hohenberger W Rödel C Wittekind C Fietkau R . Preoperative versus postoperative chemoradiotherapy for rectal cancer. N Engl J Med. (2004) 351:1731–40. doi: 10.1056/nejmoa040694 15496622

[5] Habr-Gama A Perez RO Nadalin W Sabbaga J Ribeiro U Jr Silva e Sousa AH Jr . Operative versus nonoperative treatment for stage 0 distal rectal cancer following chemoradiation therapy: long-term results. Ann Surg. (2004) 240:711–7. doi: 10.1097/01.sla.0000141194.27992.32 15383798 PMC1356472

[6] van der Valk MJM Hilling DE Bastiaannet E Meershoek-Klein Kranenbarg E Beets GL Figueiredo NL . Long-term outcomes of clinical complete responders after neoadjuvant treatment for rectal cancer in the International Watch & Wait Database (IWWD): an international multicentre registry study. Lancet. (2018) 391:2537–45. doi: 10.1016/s0140-6736(18)31078-x 29976470

[7] Garcia-Aguilar J Patil S Gollub MJ Kim JK Yuval JB Thompson HM . Organ Preservation in Patients With Rectal Adenocarcinoma Treated With Total Neoadjuvant Therapy. J Clin Oncol. (2022) 40:2546–56. doi: 10.1200/JCO.22.00032 35483010 PMC9362876

[8] Verheij FS Omer DM Williams H Lin ST Qin LX Buckley JT . Long-term results of organ preservation in patients with rectal adenocarcinoma treated with total neoadjuvant therapy: the randomized phase II OPRA trial. J Clin Oncol. (2024) 42:500–6. doi: 10.1200/jco.23.01208 37883738 PMC11578087

[9] Scott AJ Kennedy EB Berlin J Brown G Chalabi M Cho MT . Management of locally advanced rectal cancer: ASCO guideline. J Clin Oncol. (2024) 42:3355–75. doi: 10.1200/jco.24.01160 39116386

[10] Ribic CM Sargent DJ Moore MJ Thibodeau SN French AJ Goldberg RM . Tumor microsatellite-instability status as a predictor of benefit from fluorouracil-based adjuvant chemotherapy for colon cancer. N Engl J Med. (2003) 349:247–57. doi: 10.1056/NEJMoa022289 PMC3584639 12867608

[11] Sargent DJ Marsoni S Monges G Thibodeau SN Labianca R Hamilton SR . Defective mismatch repair as a predictive marker for lack of efficacy of fluorouracil-based adjuvant therapy in colon cancer. J Clin Oncol. (2010) 28:3219–26. doi: 10.1200/JCO.2009.27.1825 PMC2903323 20498393

[12] Le DT Uram JN Wang H Bartlett BR Kemberling H Eyring AD . PD-1 blockade in tumors with mismatch-repair deficiency. N Engl J Med. (2015) 372:2509–20. doi: 10.1056/nejmoa1500596 26028255 PMC4481136

[13] Le DT Durham JN Smith KN Wang H Bartlett BR Aulakh LK . Mismatch repair deficiency predicts response of solid tumors to PD-1 blockade. Science. (2017) 357:409–13. doi: 10.1126/science.aan6733 28596308 PMC5576142

[14] André T Shiu KK Kim TW Jensen BV Jensen LH Punt C . Pembrolizumab in microsatellite-instability-high advanced colorectal cancer. N Engl J Med. (2020) 383:2207–18. doi: 10.1056/NEJMoa2017699 33264544

[15] Overman MJ McDermott R Leach JL Lonardi S Lenz HJ Morse MA . Nivolumab in patients with metastatic DNA mismatch repair-deficient or microsatellite instability-high colorectal cancer (CheckMate 142): an open-label, multicentre, phase 2 study. Lancet Oncol. (2017) 18:1182–91. doi: 10.1016/s1470-2045(17)30422-9 28734759 PMC6207072

[16] André T Elez E Van Cutsem E Jensen LH Bennouna J Mendez G . Nivolumab plus ipilimumab in microsatellite-instability-high metastatic colorectal cancer. N Engl J Med. (2024) 391:2014–26. doi: 10.1056/NEJMoa2402141 39602630

[17] Chalabi M Fanchi LF Dijkstra KK Van den Berg JG Aalbers AGJ Sikorska K . Neoadjuvant immunotherapy leads to pathological responses in MMR-proficient and MMR-deficient early-stage colon cancers. Nat Med. (2020) 26:566–76. doi: 10.1038/s41591-020-0805-8 32251400

[18] Chalabi M Verschoor YL Tan PB Balduzzi S Van Lent AU Grootscholten C . Neoadjuvant immunotherapy in locally advanced mismatch repair-deficient colon cancer. N Engl J Med. (2024) 390:1949–58. doi: 10.1056/nejmoa2400634 38838311

[19] Cercek A Lumish M Sinopoli J Weiss J Shia J Lamendola-Essel M . PD-1 blockade in mismatch repair-deficient, locally advanced rectal cancer. N Engl J Med. (2022) 386:2363–76. doi: 10.1056/nejmoa2201445 35660797 PMC9492301

[20] Cercek A Foote MB Rousseau B Smith JJ Shia J Sinopoli J . Nonoperative management of mismatch repair-deficient tumors. N Engl J Med. (2025) 392:2297–308. doi: 10.1056/NEJMoa2404512 40293177 PMC12661660

[21] Boland CR Goel A . Microsatellite instability in colorectal cancer. Gastroenterology. (2010) 138:2073–2087.e3. doi: 10.1053/j.gastro.2009.12.064 20420947 PMC3037515

[22] Hause RJ Pritchard CC Shendure J Salipante SJ . Classification and characterization of microsatellite instability across 18 cancer types. Nat Med. (2016) 22:1342–50. doi: 10.1038/nm.4191 27694933

[23] Cancer Genome Atlas Network . Comprehensive molecular characterization of human colon and rectal cancer. Nature. (2012) 487:330–7. doi: 10.1038/nature11252 22810696 PMC3401966

[24] Guinney J Dienstmann R Wang X De Reyniès A Schlicker A Soneson C . The consensus molecular subtypes of colorectal cancer. Nat Med. (2015) 21:1350–6. doi: 10.1038/nm.3967 26457759 PMC4636487

[25] Angelova M Charoentong P Hackl H Fischer ML Snajder R Krogsdam AM . Characterization of the immunophenotypes and antigenomes of colorectal cancers reveals distinct tumor escape mechanisms and novel targets for immunotherapy. Genome Biol. (2015) 16:64. doi: 10.1186/s13059-015-0620-6 25853550 PMC4377852

[26] Mlecnik B Tosolini M Kirilovsky A Berger A Bindea G Meatchi T . Histopathologic-based prognostic factors of colorectal cancers are associated with the state of the local immune reaction. J Clin Oncol. (2011) 29:610–8. doi: 10.1200/jco.2010.30.5425 21245428

[27] Pagès F Mlecnik B Marliot F Bindea G Ou FS Bifulco C . International validation of the consensus Immunoscore for the classification of colon cancer: a prognostic and accuracy study. Lancet. (2018) 391:2128–39. doi: 10.1016/S0140-6736(18)30789-X 29754777

[28] Llosa NJ Cruise M Tam A Wicks EC Hechenbleikner EM Taube JM . The vigorous immune microenvironment of microsatellite instable colon cancer is balanced by multiple counter-inhibitory checkpoints. Cancer Discov. (2015) 5:43–51. doi: 10.1158/2159-8290.CD-14-0863 25358689 PMC4293246

[29] Liu J Blake SJ Yong MCR Harjunpää H Ngiow SF Takeda K . Improved Efficacy of Neoadjuvant Compared to Adjuvant Immunotherapy to Eradicate Metastatic Disease. Cancer Discov. (2016) 6:1382–99. doi: 10.1158/2159-8290.cd-16-0577 27663893

[30] Lemaire C Boileve A Manceau G Coutzac C Muller M Girot P . Neoadjuvant immunotherapy for nonmetastatic dMMR/MSI colon cancer: a real-world retrospective AGEO study. ESMO Open. (2025) 10:105516. doi: 10.1016/j.esmoop.2025.105516 40749247 PMC12337657

[31] Mirza W Khan ME Iqbal H Khan A Moeen-Ud-Din MB Khan HM . Neoadjuvant immune checkpoint inhibition in MSI-H/dMMR colorectal cancer: a systematic review of prospective trials evaluating efficacy, pathologic response, and surgical outcomes. J Gastrointest Cancer. (2025) 56:236. doi: 10.1007/s12029-025-01358-x 41343101

[32] Yu JH Xiao BY Li DD Jiang W Ding Y Wu XJ . Neoadjuvant camrelizumab plus apatinib for locally advanced microsatellite instability-high or mismatch repair-deficient colorectal cancer (NEOCAP): a single-arm, open-label, phase 2 study. Lancet Oncol. (2024) 25:843–52. doi: 10.1016/S1470-2045(24)00203-1 38852601

[33] Hu H Kang L Zhang J Wu Z Wang H Huang M . Neoadjuvant PD-1 blockade with toripalimab, with or without celecoxib, in mismatch repair-deficient or microsatellite instability-high, locally advanced, colorectal cancer (PICC): a single-centre, parallel-group, non-comparative, randomised, phase 2 trial. Lancet Gastroenterol Hepatol. (2022) 7:38–48. doi: 10.1016/s2468-1253(21)00348-4 34688374

[34] Shiu KK Jiang Y Saunders M Seligmann JF Iveson T Wilson RH . NEOPRISM-CRC: Neoadjuvant pembrolizumab stratified to tumour mutation burden for high-risk stage 2 or stage 3 deficient-MMR/MSI-high colorectal cancer. J Clin Oncol. (2024) 42:LBA3504. doi: 10.1200/JCO.2024.42.17_suppl.LBA3504 42148471

[35] Maas M Lambregts DMJ Nelemans PJ Heijnen LA Martens MH Leijtens JWA . Assessment of clinical complete response after chemoradiation for rectal cancer with digital rectal examination, endoscopy, and MRI: selection for organ-saving treatment. Ann Surg Oncol. (2015) 22:3873–80. doi: 10.1245/s10434-015-4687-9 26198074 PMC4595525

[36] Munk NE Bondeven P Pedersen BG . Diagnostic performance of MRI and endoscopy for assessing complete response in rectal cancer after neoadjuvant chemoradiotherapy: a systematic review of the literature. Acta Radiol. (2023) 64:20–31. doi: 10.1177/02841851211065925 34928715

[37] Omer DMR Williams H Lin ST Thompson HM Verheij FS Yuval JB . Diagnostic performance of endoscopy and MRI in identifying true response among patients with rectal cancer treated with total neoadjuvant therapy and selective watch and wait or TME. J Clin Oncol. (2024) 42:28. doi: 10.1200/JCO.2024.42.3_suppl.28

[38] Jayaprakasam VS Alvarez J Omer DM Gollub MJ Smith JJ Petkovska I . Watch-and-wait approach to rectal cancer: the role of imaging. Radiology. (2023) 307:e221529. doi: 10.1148/radiol.221529 36880951 PMC10068893

[39] ClinicalTrials.gov . Study of Induction PD-1 Blockade in Subjects With Locally Advanced Mismatch Repair-Deficient Solid Tumors. NCT04165772. Available online at: https://clinicaltrials.gov/study/NCT04165772 (Accessed July 21, 2026).

[40] ClinicalTrials.gov . Single Arm Study of Neoadjuvant Dostarlimab in Stage II and III Deficient Mismatch Repair Colon Cancers. NCT05239546. Available online at: https://clinicaltrials.gov/study/NCT05239546 (Accessed July 21, 2026).

[41] ClinicalTrials.gov . PD-1 Inhibitors Combined With VEGF Inhibitors for Locally Advanced dMMR/MSI-H Colorectal Cancer. NCT04715633. Available online at: https://clinicaltrials.gov/study/NCT04715633 (Accessed July 21, 2026).

[42] ClinicalTrials.gov . Neoadjuvant Pembrolizumab With a Watch-and-wait Strategy for dMMR/MSI-H Localized Colon Cancer: PREMICES Study. NCT06646445. Available online at: https://clinicaltrials.gov/study/NCT06646445 (Accessed July 21, 2026).

[43] ClinicalTrials.gov . The Efficacy of Watch and Wait Strategy or Surgery After Neoadjuvant Immunotherapy for Locally Advanced Colorectal Cancer With dMMR/MSI-H Guided by MRD Dynamic Monitoring: A Single-center, Open-label, Prospective, Phase II Clinical Trial. NCT06477991. Available online at: https://clinicaltrials.gov/study/NCT06477991 (Accessed July 21, 2026).

[44] Zhang X Cao D Du J Hu R Cai X Wu T . The efficacy of watch-and-wait strategy or surgery after neoadjuvant immunotherapy for locally advanced colorectal cancer with dMMR/MSI-H guided by ctDNA dynamic monitoring (WINDOW): a single-center, open-label, prospective, phase II study. J Clin Oncol. (2025) 43:3559. doi: 10.1200/JCO.2025.43.16_suppl.3559 42148471

[45] Dossa F Chesney TR Acuna SA Baxter NN . A watch-and-wait approach for locally advanced rectal cancer after a clinical complete response following neoadjuvant chemoradiation: a systematic review and meta-analysis. Lancet Gastroenterol Hepatol. (2017) 2:501–13. doi: 10.1016/s2468-1253(17)30074-2 28479372

[46] Smith JD Ruby JA Goodman KA Saltz LB Guillem JG Weiser MR . Nonoperative management of rectal cancer with complete clinical response after neoadjuvant therapy. Ann Surg. (2012) 256:965–72. doi: 10.1097/sla.0b013e3182759f1c 23154394

[47] Renehan AG Malcomson L Emsley R Gollins S Maw A Myint AS . Watch-and-wait approach versus surgical resection after chemoradiotherapy for patients with rectal cancer (the OnCoRe project): a propensity-score matched cohort analysis. Lancet Oncol. (2016) 17:174–83. doi: 10.1016/s1470-2045(15)00467-2 26705854

[48] Appelt AL Pløen J Harling H Jensen FS Jensen LH Jørgensen JCR . High-dose chemoradiotherapy and watchful waiting for distal rectal cancer: a prospective observational study. Lancet Oncol. (2015) 16:919–27. doi: 10.1016/s1470-2045(15)00120-5 26156652

[49] Tie J Wang Y Tomasetti C Li L Springer S Kinde I . Circulating tumor DNA analysis detects minimal residual disease and predicts recurrence in patients with stage II colon cancer. Sci Transl Med. (2016) 8:346ra92. doi: 10.1126/scitranslmed.aaf6219 27384348 PMC5346159

[50] Kotani D Oki E Nakamura Y Yukami H Mishima S Bando H . Molecular residual disease and efficacy of adjuvant chemotherapy in patients with colorectal cancer. Nat Med. (2023) 29:127–34. doi: 10.1038/s41591-022-02115-4 36646802 PMC9873552

[51] Salama MM Eddershaw C Temperley HC Perthiani AK Larkin JO Mehigan BJ . Circulating tumour DNA after neoadjuvant therapy in non-metastatic colon cancer: a systematic review and implications for surgical decision-making. Cancers (Basel). (2026) 18:815. doi: 10.3390/cancers18050815 41827748 PMC12984180

[52] Schneider BJ Naidoo J Santomasso BD Lacchetti C Adkins S Anadkat M . Management of immune-related adverse events in patients treated with immune checkpoint inhibitor therapy: ASCO guideline update. J Clin Oncol. (2021) 39:4073–126. doi: 10.1200/jco.21.01440 34724392

[53] Haanen J Obeid M Spain L Carbonnel F Wang Y Robert C . Management of toxicities from immunotherapy: ESMO Clinical Practice Guideline for diagnosis, treatment and follow-up. Ann Oncol. (2022) 33:1217–38. doi: 10.1016/j.annonc.2022.10.001 36270461

[54] Nguyen TH Chokshi RV . Low anterior resection syndrome. Curr Gastroenterol Rep. (2020) 22:48. doi: 10.1007/s11894-020-00785-z 32749603 PMC8370104

[55] Rosen H Sebesta CG Sebesta C . Management of Low Anterior Resection Syndrome (LARS) Following Resection for Rectal Cancer. Cancers (Basel). (2023) 15:778. doi: 10.3390/cancers15030778 36765736 PMC9913853

[56] Custers PA van der Sande ME Grotenhuis BA Peters FP van Kuijk SMJ Beets GL . Long-term quality of life and functional outcome of patients with rectal cancer following a watch-and-wait approach. JAMA Surg. (2023) 158:e230146. doi: 10.1001/jamasurg.2023.0146 36988922 PMC10061319

[57] Basch E Deal AM Dueck AC Scher HI Kris MG Hudis C . Overall survival results of a trial assessing patient-reported outcomes for symptom monitoring during routine cancer treatment. JAMA. (2017) 318:197–8. doi: 10.1001/jama.2017.7156 28586821 PMC5817466

[58] Calvert M Blazeby J Altman DG Revicki DA Moher D Brundage MD . Reporting of patient-reported outcomes in randomized trials: the CONSORT PRO extension. JAMA. (2013) 309:814–22. doi: 10.1001/jama.2013.879 23443445

[59] Williams CJM Peddle AM Kasi PM Seligmann JF Roxburgh CS Middleton GW . Neoadjuvant immunotherapy for dMMR and pMMR colorectal cancers: therapeutic strategies and putative biomarkers of response. Nat Rev Clin Oncol. (2024) 21:839–51. doi: 10.1038/s41571-024-00943-6 39317818

[60] Vasen HFA Blanco I Aktan-Collan K Gopie JP Alonso A Aretz S . Revised guidelines for the clinical management of Lynch syndrome (HNPCC): recommendations by a group of European experts. Gut. (2013) 62:812–23. doi: 10.1136/gutjnl-2012-304356 23408351 PMC3647358

[61] Umar A Boland CR Terdiman JP Syngal S de la Chapelle A Rüschoff J . Revised Bethesda Guidelines for hereditary nonpolyposis colorectal cancer (Lynch syndrome) and microsatellite instability. J Natl Cancer Inst. (2004) 96:261–8. doi: 10.1093/jnci/djh034 PMC2933058 14970275

[62] Seppälä TT Latchford A Negoi I Sampaio Soares A Jimenez-Rodriguez R Sánchez-Guillén L . European guidelines from the EHTG and ESCP for Lynch syndrome: an updated third edition of the Mallorca guidelines based on gene and gender. Br J Surg. (2021) 108:484–98. doi: 10.1002/bjs.11902 34043773 PMC10364896

[63] Tanakaya K Yamaguchi T Hirata K Yamada M Kumamoto K Akiyama Y . Japanese Society for Cancer of the Colon and Rectum (JSCCR) guidelines 2024 for the clinical practice of hereditary colorectal cancer. Int J Clin Oncol. (2026) 31:1–66. doi: 10.1007/s10147-025-02892-1 41214301 PMC12770061

[64] Collins GS Reitsma JB Altman DG Moons KGM . Transparent reporting of a multivariable prediction model for Individual Prognosis or Diagnosis (TRIPOD): the TRIPOD statement. Ann Intern Med. (2015) 162:55–63. doi: 10.7326/M14-0697 25560714

[65] ClinicalTrials.gov . A Study of Dostarlimab in Untreated Dmmr/MSI-H Locally Advanced Rectal Cancer (AZUR-1). Available online at: https://clinicaltrials.gov/study/NCT05723562 (Accessed July 21, 2026).

[66] GSK plc . Jemperli (dostarlimab) achieves sustained clinical complete responses in dMMR/MSI-H locally advanced rectal cancer (2026). Available online at: https://www.gsk.com/en-gb/media/press-releases/jemperli-dostarlimab-achieves-sustained-clinical-complete-responses-in-dmmrmsi-h-locally-advanced-rectal-cancer/ (Accessed July 21, 2026).

[67] ClinicalTrials.gov . Pd1 Antibody Sintilimab ± Chemoradiotherapy for Locally Advanced Rectal Cancer. Available online at: https://clinicaltrials.gov/study/NCT04304209 (Accessed July 21, 2026).

[68] ClinicalTrials.gov . Neoadjuvant Tisleizumab(BGB-A317) for dMMR/MSI-H Non-late Stage CRC Patients Before Surgery. Available online at: https://clinicaltrials.gov/study/NCT06262581 (Accessed July 21, 2026).

