# Supplementary material for: Organ preservation after neoadjuvant immunotherapy in mismatch repair-deficient colorectal cancer: response assessment, watch-and-wait, and salvage surgery
Source: Front Oncol. 2026 Aug 25;16:1908199. doi: 10.3389/fonc.2026.1908199 (PMC13551423; doi:10.3389/fonc.2026.1908199)
Supplement: Supplementary file 1 [file Table1.docx]

**Supplementary Material**

**Supplementary Table S1. Major clinical studies of neoadjuvant immunotherapy in dMMR colorectal cancer**

| **Study** | **Cancer type** | **Regimen** | **Population** | **Response endpoint** | **Surgery performed or omitted** | **Follow-up** | **Key implication** |
| --- | --- | --- | --- | --- | --- | --- | --- |
| NICHE Chalabi et al., Nat Med 2020 (17) | Early-stage colon cancer | Nivolumab plus ipilimumab (short-course preoperative dual checkpoint blockade) | Treatment-naive, resectable colon cancer; exploratory dMMR and pMMR cohorts | Pathological response; pCR/MPR assessed in the resection specimen | Surgery performed as planned | Short preoperative-window study; early follow-up | First proof-of-concept that dMMR colon cancers can undergo rapid immune-mediated pathological regression before surgery. |
| PICC Hu et al., Lancet Gastroenterol Hepatol 2022 (33) | Locally advanced colorectal cancer | Toripalimab with or without celecoxib before surgery | dMMR/MSI-H, cT3-4 or node-positive, resectable colorectal cancer | pCR as primary endpoint; MPR also reported | All patients underwent surgical resection; no treatment-related surgical delay was reported | Median follow-up about 15 months at initial report | Supported high activity of neoadjuvant single-agent PD-1 blockade and explored whether COX-2 inhibition may further enhance response. |
| Dostarlimab rectal cancer Cercek et al., NEJM 2022 (19) | Locally advanced rectal cancer | Single-agent dostarlimab every 3 weeks for 6 months | dMMR, stage II/III rectal cancer planned for standard chemoradiotherapy and surgery if incomplete response | cCR by DRE, endoscopy, MRI, FDG-PET/CT and biopsy when appropriate | Surgery and chemoradiotherapy omitted in patients with cCR during reported follow-up | Early follow-up; longer-term durability required | Shifted the field from “immunotherapy before surgery” to potential nonoperative management after robust cCR in dMMR rectal cancer. |
| NICHE-2 Chalabi et al., NEJM 2024 (18) | Locally advanced colon cancer | Nivolumab plus ipilimumab (short-course neoadjuvant regimen) | Nonmetastatic locally advanced dMMR colon cancer | Safety and timely surgery; pathological response, MPR and pCR | Surgery performed in nearly all patients as planned | Median follow-up exceeded 2 years in the published report | Confirmed feasibility and very high pathological response rates, but did not establish routine omission of colectomy. |
| NEOPRISM-CRC Shiu et al., JCO 2024 abstract (34) | High-risk stage II/III colorectal cancer | Pembrolizumab stratified by tumor mutational burden; short preoperative course | Operable high-risk stage II or III dMMR/MSI-H colorectal cancer | pCR as primary endpoint; translational biomarkers including TMB and ctDNA explored | Surgery planned after neoadjuvant pembrolizumab | Immature follow-up at meeting report | Provided multicenter support for short-course PD-1 blockade before surgery and highlighted biomarker-adaptive trial design. |
| Nonoperative dMMR tumors Cercek et al., NEJM 2025 (20) | dMMR rectal cancer and other early-stage dMMR tumors | Single-agent dostarlimab | Early-stage dMMR tumors; rectal cancer cohort and nonrectal cohort with residual disease directed to surgery | Sustained cCR and recurrence-free survival; cCR guided nonoperative management | Nonoperative management elected after cCR; resection intended for residual disease | Median follow-up for recurrence about 20 months in the full analysis | Strengthened the concept that complete clinical regression after PD-1 blockade can support structured nonoperative management, while preserving surgery for nonresponders. |

**Abbreviations:** cCR, clinical complete response; ctDNA, circulating tumor DNA; dMMR, mismatch repair-deficient; DRE, digital rectal examination; FDG-PET/CT, fluorodeoxyglucose positron emission tomography/computed tomography; MPR, major pathological response; MRI, magnetic resonance imaging; MSI-H, microsatellite instability-high; pCR, pathological complete response; pMMR, mismatch repair-proficient; TMB, tumor mutational burden.

**Note:** This table is intended for a narrative review and summarizes trial-level implications rather than serving as a cross-trial efficacy comparison, because study populations, regimens, endpoints, and follow-up durations differ across studies.

**Supplementary Table S2. Proposed surveillance framework during watch-and-wait after immunotherapy**

| **Time after cCR** | **DRE** | **Endoscopy** | **MRI** | **CT** | **CEA** | **ctDNA optional** | **Clinical action** |
| --- | --- | --- | --- | --- | --- | --- | --- |
| At cCR confirmation / entry to W&W | Document no palpable residual tumor. | Confirm flat scar or mucosal normalization; biopsy only if clinically indicated. | Pelvic MRI with DWI; exclude residual wall signal and suspicious nodes. | Chest/abdomen/pelvis CT for systemic restaging. | Baseline post-treatment value. | Optional baseline sample for longitudinal comparison. | Enter W&W only after MDT confirmation of cCR, patient consent, and surveillance feasibility. |
| Years 0-2 | Every 3-4 months. | Every 3-4 months. | Every 3-6 months, preferably with DWI. | Every 6-12 months. | Every 3-6 months. | Every 3-6 months if available; interpret with imaging and endoscopy. | Continue W&W if cCR is sustained; repeat assessment promptly for equivocal findings; restage and consider salvage surgery for regrowth. |
| Years 3-5 | Every 6 months. | Every 6 months, then annually if stable. | Every 6-12 months. | Annually or risk-adapted. | Every 6 months. | Every 6-12 months if used. | Maintain structured surveillance; investigate new symptoms, rising markers, positive ctDNA, or suspicious imaging/endoscopic change. |
| After 5 years | Annually or symptom-driven. | Annually or per survivorship/colonoscopy schedule. | Risk-adapted; consider annual MRI in high-risk patients. | Per survivorship guidelines and risk profile. | Annually or risk-adapted. | Optional; individualized. | Transition to survivorship care while maintaining rapid re-entry to MDT review for any suspicious finding. |
| Triggered reassessment at any time | Immediate examination if bleeding, pain, altered bowel habits, or abnormal imaging occurs. | Urgent endoscopy and targeted biopsy if suspicious mucosal change is present. | Repeat MRI to characterize wall, mesorectal, or nodal abnormality. | Systemic restaging if local regrowth or molecular/radiologic relapse is suspected. | Repeat if rising or discordant. | Confirm with repeat testing when positive or discordant; do not act in isolation. | Discuss in MDT; proceed to salvage surgery or protocol-defined treatment when residual disease or regrowth is convincing. |

**Abbreviations:** cCR, clinical complete response; DRE, digital rectal examination; W&W, watch-and-wait; MRI, magnetic resonance imaging; DWI, diffusion-weighted imaging; CT, computed tomography; CEA, carcinoembryonic antigen; ctDNA, circulating tumor DNA; MDT, multidisciplinary team. This schedule is illustrative and should be individualized according to baseline stage, response confidence, institutional expertise, and **patient adherence.** This framework is an illustrative synthesis based on available rectal cancer surveillance literature (6, 7, 35–38, 45–48) and has not been prospectively validated specifically after neoadjuvant immunotherapy; it should not be interpreted as a formal guideline.
